# Management of Ocular Trauma in Emergency (MOTE) Trial: A pilot randomized double-blinded trial comparing topical amethocaine with saline in the outpatient management of corneal trauma

**DOI:** 10.4103/0974-2700.44676

**Published:** 2009

**Authors:** Joseph Y S Ting, Kenneth J Barns, John L Holmes

**Affiliations:** Department of Emergency Medicine, Mater Adults' Hospital, Raymond TCE, South Brisbane, 4101 Qld, Australia

**Keywords:** Corneal injury, ocular anesthetic, topical analgesia

## Abstract

**Background:**

It is unclear whether local anesthetic eye drops can be safely used for the topical anesthesia of patients with minor corneal injury who are discharged from the emergency department (ED).

**Objectives:**

To assess whether topical 0.4% amethocaine self-administered to a maximum recommended frequency of once every hour for 36–48 h is safe in the management of uncomplicated corneal injury in patients discharged from the ED.

**Patients and Methods:**

A pilot randomized double-blinded trial comparing topical 0.4% amethocaine with topical normal saline.

**Results:**

Forty-seven subjects were recruited, with 22 randomized to receive amethocaine and 25 to receive placebo (normal saline). Baseline characteristics, including corneal injury type, were similar in both groups. There were no significant functional or clinical adverse sequelae in the majority of enrolled patients who could be contacted at 2 weeks (17/22 for amethocaine and 21/25 for placebo). Follow-up for the primary study outcome was suboptimal, with only 7/22 from the amethocaine group and 9/25 from the saline group presenting for 36–48 h review; there was a statistically nonsignificant trend for persistence of the corneal defect in the amethocaine group as compared with the saline group (2/7 and 1/9, respectively).

**Conclusion:**

Compared with saline drops, amethocaine eye drops are not definitely safe but they are effective for topical analgesia in minor corneal injury. Until further definitive studies, topical nonsteroidal agents or long-lasting artificial tears may be preferred for the topical analgesia of minor corneal injury. Return for corneal re-evaluation will necessarily remain suboptimal in an otherwise self-limiting condition, leading to a bias even if study recruitment is good.

Corneal abrasion, with or without retained foreign body, is the most frequent ophthalmic presentation to an emergency department (ED).[1] Although usually self-limiting, corneal abrasions can cause severe pain during the first 24 h. This pain may not be adequately controlled by oral analgesics.[2]

Trials investigating the outpatient management of corneal abrasions suggest that topical ophthalmic preparations of non-steroidal anti-inflammatory drugs (NSAIDs) are effective for subjective pain relief and possess short-term safety. However, ED use of these agents is limited because the anesthesia achieved with topical NSAIDs, while statistically significant, did not reach clinically important levels when measured using visual analog pain scores in these trials;[3] further, topical NSAIDs may lead to significant adverse events in high doses, with prolonged use, or when there are ocular co-morbidities.[4] These concerns prompted the recall of 0.1% diclofenac ophthalmic solution in the United States in 1999.[5]

There is a therefore a need for a safe and effective method for managing the pain of corneal abrasions and ultraviolet keratitis. Ophthalmic topical anesthetics are known to provide excellent corneal anesthesia for 15–20 min, with return of normal sensation within 60 min.[6–8]

Despite its established role in facilitating assessment and instrumentation of the cornea during eye examination or surgery, there has been little research into the use of topical anesthetics in the outpatient setting and then only in post-surgical patients. When compared with placebo, local anesthetics have been shown reduce ocular pain without affecting corneal wound healing or visual performance in post-surgical patients for 24 h after hospital discharge.[9,10] However, the use of topical anesthetics for corneal abrasions after discharge is widely discouraged[11]; there have been case reports of complications related to neurotrophic corneas (e.g., unrecognized worsening of the corneal injury, secondary infection, etc.), delayed healing with persistent epithelial defects, cytotoxic damage to the corneal epithelium, corneal hyperesthesia, and even loss of visual acuity, especially with prolonged or excessive use.[12] There is therefore a widely held belief among clinicians that self-administration of topical ocular anesthesia is hazardous.[11]

There is no clinical trial data to suggest that a 1- to 3-day course of intermittent self-administered topical anesthesia would cause damage to the eye in the post-surgical setting. Several trials[9,10] have demonstrated that topical anesthetics effectively relieve pain during the first 3 days after keratectomy without causing side effects or delayed healing.

However, it remains unclear whether this safety profile in the post-surgical setting would extend to patients with minor uncomplicated corneal injury. Therefore, we conducted a study to assess whether use of topical anesthetics during the first 36–48 h after initial ED assessment of a corneal abrasion or ultraviolet keratitis reduces eye pain and increases patient satisfaction without a clinically significant delay in epithelial healing or other complications.

## PATIENTS AND METHODS

We conducted a randomized double-blinded trial comparing self-administered topical 0.4% amethocaine with 0.9% saline solution in the outpatient management of uncomplicated corneal injury. The study received approval from the Mater Health Services Human Research and Ethics Committee and is registered with the Australian Clinical Trials Registry (ACTRN012605000273684).

Recruitment occurred over 12 months at the Mater Adult Hospital ED, an urban ED with specialist ophthalmology services. For inclusion in the study patients had to have any one of the following: (i) traumatic superficial corneal abrasion(s), (ii) superficial corneal abrasion(s) with retained foreign body(s), or (iii) keratitis from welding flash exposure.

Exclusion criteria included the following: more than 36 h elapsed since the event causing the corneal injury, patient less than 18 years old, history of adverse reaction to any local anesthetic agent or to any ophthalmic preparation, eye pathology (e.g., diabetic retinopathy, glaucoma) other than refractive error, contact lens use, current pregnancy or lactation, signs of conjunctival infection, functionally one-eyed, and patient requiring urgent referral to ophthalmology (including penetrating eye injury).

**Figure 1:**
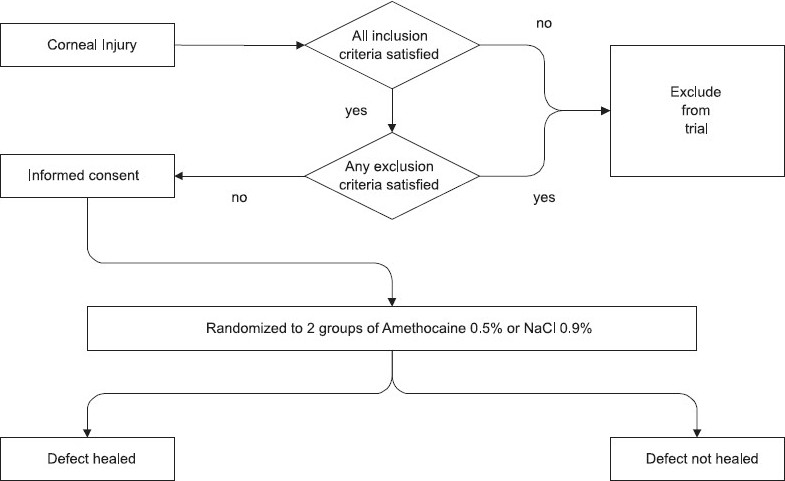
Management of Ocular Trauma in Emergency Trial: recruitment and follow-up for primary outcome of corneal healing.

Eligible patients were approached for informed consent [Figure 1].

For the purposes of standardization, no patient in the trial was given cycloplegics nor was eye patching applied; otherwise, initial ED assessment and management was at the treating doctor's discretion.

Data regarding corneal healing was excluded for patients presenting for review at < 36 or > 48 h after recruitment, which is less or more than the duration expected for re-epithelization. Patients with a rust ring requiring further instrumentation at 36–48 h review were also excluded from the statistical analysis since it would not be possible to determine whether the patient's symptoms were ascribable to the treatment he or she was allocated or to the retained foreign body.

Mater research pharmacists not involved in the design or conduct of the study prepared identical, clear, minim packs of either 0.9% saline solution or 0.4% amethocaine solution. There were 6 batches, with each batch containing 16 randomly ordered mimim packs (8 saline and 8 amethocaine packs); the batches were sent to the ED in no particular order. The mimim packs in the 6 batches were sequentially numbered from 1 to 96 (6 × 16 = 96). After the patient had given informed consent, he or she was given the next available minim pack, the composition of which was not known to either the patient or clinician. The patient was instructed to instill one drop in the affected eye(s) once hourly as needed for pain relief and to take oral analgesia as required. The containers were stored in a drug refrigerator.

Data was collected at original assessment in the ED; during ED review 36–48 h after recruitment; in the form of a diary maintained by the patient recording visual analogue pain scores; and telephone follow-up interview 2 weeks after recruitment. Intention-to-treat analysis was performed for all outcomes.

The primary outcome was healing of the corneal defect; potential delay in healing is the main concern with the use of outpatient topical anesthesia. We compared two independent proportions (amethocaine vs. saline) of patients whose cornea had completely re-reepithelialized at 36–48 h. Complete re-epithelialization was defined as the absence of fluorescein stain uptake. Sequential analysis in the context of the triangular test[13] was used to determine any significant difference between amethocaine and saline for the primary outcome.

Secondary outcomes were assessed at the 2-week telephone interview; these outcomes included use of oral analgesia, unscheduled medical review, visual problems, and satisfaction with treatment received. A patient diary was used to record third hourly patient-rated pain perception; pain was measured using a visual analogue scale – an ungraded 100-mm horizontal line with the left end indicating “No pain” and the right end “Worst pain imaginable.” The patient was asked to apply a vertical mark on the line to indicate the severity of the pain. One of the investigators measured the distance in millimeters from the left (“No pain”) end of the line. Cumulative scores were a summation of up to 12 pain measurements over 36 h. A chi square comparison of two independent proportions for binary secondary outcomes (oral analgesia use, unscheduled medical review, visual problems, and patient satisfaction) was performed. We used the Student's t-test to compare the difference between the mean cumulative pain scores in the two treatment groups.

Power and sample size calculations were based on a two-tailed difference of 25–50%, as there has been a large variation in corneal healing rates in previous studies.[14,15] This trial may change clinical practice if it demonstrates equivalence in healing rates between local anesthesia and saline—in other words, if it fails to demonstrate that there is a difference between the two groups. For that reason, it is important to increase the power of the trial and acceptable to relax the tolerance for a type I error.[16] Assuming a type I (α) error rate of 0.2 and a power of 0.95, the maximum number of cases in a two-group trial is 237 based on a Triangular Test.[13] As this is a “worst-case scenario” sample size, we decided that sequential analysis would be carried out after each batch of 16 patients. If the actual difference turned out to be larger or smaller than expected, we would be able to come to a conclusion with a smaller sample and the trial could be completed earlier.

## RESULTS

Baseline characteristics were similar in the amethocaine and saline groups [Table 1].

**Table 1:** Baseline characteristics of the 47 patients randomized

	Treatment	Saline
Number of patients	22	25
Mean age (years)	35.1	33.6
Male sex	22 (100)	25 (100)
Mean time from injury (hours)	13.8	15.8
Injury type		
Corneal abrasion	8 (36)	7 (28)
Corneal foreign body	9 (41)	11 (44)
Welding flash burn	4 (18)	6 (24)
Welding flash burn and corneal foreign body	1 (5)	1 (4)
Topical antibiotics on discharge	8/20 (40)	8/18 (44)

FIGURES IN PARENTHESIS INDICATE PERCENTAGE

**Figure 2:**
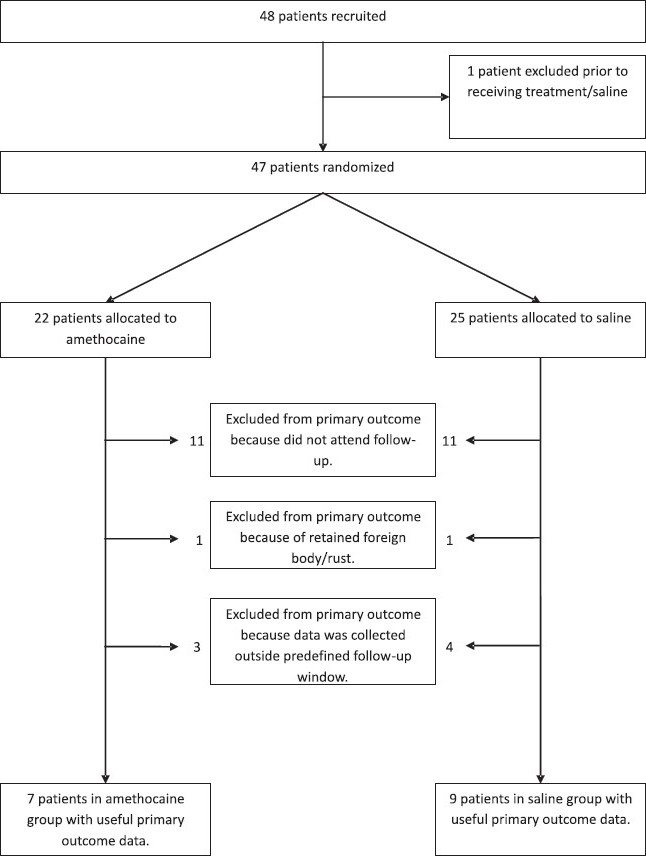
Flow diagram of patients recruited into the MOTE Trial.

Figure 2 gives an account of the patient flow through the study. Sixteen (7 amethocaine, 9 saline), or 34% of the 47 randomized patients, had valid primary outcome data; secondary outcome data was available for 38 patients (17 amethocaine, 21 saline; 83%).

There was a statistically nonsignificant trend for persistence of the corneal defect in the amethocaine group as compared with saline group (2/7 vs. 1/9) [Table 2].

**Table 2:** Comparison of primary and secondary outcomes between topical amethocaine and saline

	Amethocaine	Saline	*P*
Primary outcome			
Corneal defect at 36–48 h	2/7 (29)	1/9 (11)	0.55
Secondary outcomes (“between 48 h and 2 weeks after initial assessment, was there…”)			
Review by another health practitioner	2/17 (12)	2/21 (10)	1
Analgesia used for eye pain	0/17	0/21	–
Visual problems	3/17 (18)	1/21 (5)	0.307
Satisfaction with management	17/17 (100)	21/21 (100)	–

FIGURES IN PARENTHESIS INDICATE PERCENTAGE

At 2 weeks, the satisfaction with their management was similar in both groups and there was no significant difference between the groups in the amount of oral analgesia used for eye pain, in ongoing visual problems, or in the need for unscheduled medical review.

Twelve of 22 patients (55%) allocated to the amethocaine group and 9 of 25 (36%) allocated to the saline group completed pain diaries. Those receiving saline had a higher mean pain burden of 629 ± 172 mm (mean ± standard deviation) than those receiving amethocaine (404 ± 75 mm); this could not be statistically tested because only a small number of diaries were returned.

## DISCUSSION

Corneal abrasions, foreign bodies, and welding flash burns can cause severe pain that may not be controlled by oral analgesia for up to 48 h.[2] The outpatient use of topical ocular anesthetics in this setting has been discouraged because of case reports of complications related to an anesthetic cornea, including worsening of corneal injury, delayed healing, and secondary infection.[12] However, when used for 24 h following photorefractive keratectomy, topical anesthetics have been effective in reducing pain, without impairing corneal healing or visual performance.[9,17]

We performed a randomized, double-blinded, saline -controlled comparison trial to determine whether using amethocaine 0.4% eye drops hourly as needed for up to 48 h following uncomplicated corneal injury is a safe and effective method of analgesia as compared with topical saline. Persisting corneal defect was slightly more likely at 36–48 h in the amethocaine group than in the saline group, although the numbers were small (2/7 vs. 1/9). However, the secondary outcomes—oral analgesia used for eye pain, ongoing visual problems, need for unscheduled medical review, and patient satisfaction—did not differ between the two groups.

Mean cumulative eye pain scores was lower for the amethocaine group. A high proportion (83%) of subjects could be followed up by telephone interview and the results suggest that amethocaine causes no clinically significant complications at 2 weeks. However, a formal ED eye assessment with quantification of the healing of the epithelial defect, rather than just information obtained through a telephone interview, would have been more reliable.

Amethocaine was effective in reducing ocular pain compared with saline in this study; it conferred an analgesic benefit without causing any significant adverse sequelae at 2 weeks. This is the first published study evaluating the outpatient use of topical anesthesia following uncomplicated corneal injury, but there were several limitations in its conduct. The self-limiting nature of minor uncomplicated corneal injury reduces patient inclination to attend follow-up; in addition to leading to bias from loss to follow-up, this reduces the power of the study to detect any delay in corneal healing. Only patients with minor corneal injury expected to heal well within the 36–48 h follow-up period of the study were eligible for the study; the investigators did not attempt to impose the theoretical risk associated with a poorly sensate cornea on patients with corneal injury of higher severity that require a longer period to re-epithelize.

The size of the corneal defect at recruitment and follow-up was not assessed as sophisticated ophthalmological assessment skills and the equipment required are usually not available in an ED. This situation would be expected at most non–eye hospital EDs and the simple practical slit-lamp assessment used in the study should render its findings more generalizable. However, this also meant that standardized evaluation of the rate of corneal healing was not possible, reducing the validity of comparison of corneal defect size between, and within the same, enrolled subjects due to interobserver and inter-event variability.

There was no difference between the majority of amethocaine- and saline-treated subjects who could be contacted at 2 weeks (17/22 for amethocaine; 21/25 for saline) with respect to clinically or functionally significant adverse sequelae. However, it remains inconclusive whether dispensing topical anesthetics to patients discharged from the ED, who are otherwise unable to control pain following uncomplicated corneal injury, could potentially delay corneal re-epithelization, as only 34% (16 of 47) of the randomized subjects returned for follow-up at 36–48 h. This drop-out rate was unavoidable; on being contacted after failure to attend follow-up, most subjects cited insignificant pain or no pain and absence of visual problems as the main reasons for noncompliance.

Although topical anesthesia has been found to be both safe and effective in the treatment of post-keratectomy pain,[9,17] there have been several case reports of keratopathy related to topical anesthetic overuse or abuse.[18–20] However, there has been no published literature on topical anesthesia use after uncomplicated minor corneal trauma.

As there was a statistically nonsignificant trend for persistence of corneal defect in the amethocaine group compared with the saline group (2/7 vs. 1/9), amethocaine needs to be used with caution in minor corneal injury. The increased risk of secondary corneal injury due to a poorly sensate cornea is compounded by poor compliance with attendance at review; there is also the possibility that the amethocaine drops will be stored for future unsupervised use. Although the majority of subjects in this study had no adverse sequelae at the 2-week telephone interview, two-thirds did not return for corneal assessment at 36–48 h.

Loss to 36–48 h follow-up for a self-limiting condition such as uncomplicated corneal injury will remain a problem in any future study; higher numbers of subjects enrolled will only partly compensate for the suboptimal adherence to follow-up and the inherent bias resulting from the latter. There is a need for an adequately powered trial, with less follow-up attrition, to conclusively confirm or refute the common belief[11] that topical anesthetics are harmful when used by outpatients, specifically with regard to delayed corneal healing.

Compared with saline drops, amethocaine eye drops are probably not definitely safe but they are effective for topical analgesia in minor corneal injury. Until further definitive study, topical non-steroidal agents or long-lasting artificial tears may be preferred for the topical analgesia of minor corneal injury. Return for corneal re-evaluation will necessarily remain suboptimal in this self-limiting condition, leading to a bias even if study recruitment is good. Researchers conducting future studies may have to consider offering incentives such as reimbursement for expenses incurred in complying with follow-up or other strategies to reduce loss to follow-up.

### Opportunities for future research

This small study suggests that at 2 weeks, there are no significant functional or clinical adverse sequelae in patients with minor corneal injury who were allocated topical 0.4% amethocaine. Such indirect evidence of safety would justify replicating this study, with improved recruitment and compliance for follow-up review. Treatment-related delay in corneal healing could then be more reliably assessed.

## CONCLUSIONS

We have conducted the first published randomized controlled trial to assess whether topical 0.4% amethocaine self-administered to a maximum recommended frequency of once every hour for 36–48 h is safe compared with topical saline in the management of patients with uncomplicated corneal injury who are discharged after assessment in the ED. However, it remains uncertain whether a brief period of patient self-administered topical anesthesia is safe to use for alleviating the acute pain resulting from corneal injury. Although no adverse effects were encountered at 2 weeks, amethocaine eye drops are not definitely safe in terms of healing of the corneal defect at 36–48 h, but they are effective for topical analgesia in minor corneal injury. Until further definitive study, topical non-steroidal agents or long-lasting artificial tears may be preferred for the topical analgesia of minor corneal injury. Return for corneal re-evaluation will necessarily remain suboptimal in this self-limiting condition, potentially leading to a bias even if study recruitment is good.
